# Thinking Outside the (Enrollment) Box: “Who, disguised as a Public Educator, . . .”

**DOI:** 10.1093/geroni/igaa057.1756

**Published:** 2020-12-16

**Authors:** Roger Anunsen

**Affiliations:** Portland Community College, Portland, Oregon, United States

## Abstract

“Thank you, I really enjoyed your talk. I understand you teach college classes and I’m wondering how . . .” That’s what can happen when a “College Educator” steps off campus, steps into the community. That’s the enrollment superpower of a “Public Educator.” We’ll track a decade of innovative off-campus educational presentations strategically positioned to target new students, lead to cognitive-enhancing programs with adult residential living communities, and, importantly, help reset the role and relevancy of today’s gerontology faculty. Examples will include service club meetings, public agency, business and non-profit training retreats, and residential community staff training. Examples include “This is Your Brain on . . .” events such as Music, Loneliness, Sleep, Volunteering, Sports Fandom, Quilting, Golf and, of course, TasteAerobics featuring Brain-Healthy Chocolate. We’ll conclude with an innovative enrollment and awareness-raising project in a small city west of Portland that kicked off with a 6-part Aging Education Series. Part of a symposium sponsored by the Community College Interest Group.

